# Effects of Physical Training on Physical and Psychological Parameters in Individuals with Patella Tendinopathy: A Systematic Review and Meta-Analysis

**DOI:** 10.3390/sports9010012

**Published:** 2021-01-19

**Authors:** Marc Niering, Thomas Muehlbauer

**Affiliations:** 1 Department of Health and Social Affairs, FHM Hannover—University of Applied Sciences, 30163 Hannover, Germany; 2 Division of Movement and Training Sciences/Biomechanics of Sport, University of Duisburg-Essen, 45141 Essen, Germany; thomas.muehlbauer@uni-due.de

**Keywords:** patellar tendinosis, tendinitis, tendinopathy, athletes, physical fitness, intervention

## Abstract

The effectiveness of physical training on physical and psychological parameters in individuals with patella tendinopathy has not been investigated in a systematic review and meta-analysis. The aim of the present study was to determine the effects of physical exercise interventions for measures of physical and psychological performance in subjects with patella tendinopathy. A computerised systematic literature search was conducted in the electronic databases PubMed, Medline, and Web of Science from January 1960 to July 2020. Initially, 506 articles were identified for review of which eleven articles met the inclusion criteria. Our results revealed a small effect (weighted mean standardized mean difference (SMD) = 0.12; nine studies) of physical training on the psychological measure Victorian Institute of Sport Assessment–Patellar tendon scale and a medium effect (weighted mean SMD = 0.61; five studies) on the psychological measure visual analogue scale—both in favour of the intervention group. In contrast, a small effect (weighted mean SMD = −0.05; two studies) in favour of the control group was detected for the physical measure muscle power. Compared to the control condition, physical training seems to be an effective means to improve psychological but not physical parameters in individuals with patella tendinopathy; although conclusions on the latter could have been biased by the small amount of eligible studies (*n* = 2). In addition, the predetermined cut-off value of ≥6 for the Physiotherapy Evidence Database scale score (i.e., assessment of methodological quality) was only achieved by six out of eleven studies. Thus, further research of high methodological quality is needed to verify whether there is or is not an effect of physical training on physical parameters in persons with patella tendinopathy.

## 1. Introduction

Overuse injuries affect adolescent athletes with incidence rates up to 50% [1]. Patella tendinopathy, also known as jumper’s knee (JK), is a common overload injury that is frequently observed in young athletes performing high-impact jumping sports, such as soccer, volleyball, or basketball [2,3,4]. Compared to other common injuries (e.g., anterior cruciate ligament lesions), the prevalence of patella tendinopathy is relatively high affecting approximately 31.9% of basketball players and 44.6% in volleyball players [5], which underlines the relevance of this injury among common sports. Especially within a competitive season, during which athletes are under constant pressure to perform on a high level, treatment of patella tendinopathy can be challenging [6]. Male athletes are affected twice as often as females [7]. Age does not play a significant role with regard to the presentation of symptoms [3].

A variety of extrinsic (e.g., high tendon loads due to exercise or short recovery periods after mechanical overload) and intrinsic factors (e.g., malalignment, muscular tightness, imbalance) have been shown to cause patella tendinopathy [8]. Direct medical expenses of patients with tendinopathy are around £20,000 to £30,000 (currency in 2015) and are comparable to the medical expenses of osteoarthritis and osteoporosis (currency in 2005 and 2004, respectively) [9]. Recent literature reports a loss of work ability by 16%, as well as a 36% decrease in productivity in patients with patellar tendinopathy [10], both adding to the indirect medical costs.

Several treatment options aim to reduce pain related to patella tendinopathy. Apart from surgical interventions, dry needling, extracorporeal shockwave therapy, and a wide range of physical exercise interventions, which have proven useful in the reduction of pain and symptoms, are available. Pain relief interventions using physical training present many benefits for patients. Exercises that improve symptoms of patella tendinopathy and prevent consequential trauma can be practiced with lower pain intensity, therefore improving execution of these exercise [11]. More specifically, eccentric muscle strengthening has proven to be particularly effective when performed on a declined surface [12,13]. Also, a heavy slow resistance training program showed a significant reduction of tendon stiffness and similar effects in treating patella tendinopathy [14]. The main effects of the aforementioned exercise therapies are a reduction in pain with and without stress on the tendon, as well as improved athletic ability, in particular jump height. However, the current literature is sparse with regard to psychological parameters, although pain relief after a long period of injury-related downtime often correlates with higher motivation in sports and everyday activities. The aim of this systematic review and meta-analysis was to determine the effects of physical training on physical and psychological parameters in individuals with patella tendinopathy. Due to the high prevalence of patella tendinopathy in adolescent athletes in particular [4], it is of utmost importance to measure the effectiveness of physical exercise on both outcome categories. More precisely, there is evidence that better values in physical and psychological parameters have an impact on athletes’ performance during exercise and competition [15,16].

## 2. Methods

In the present study, we followed the recommendations of the Preferred Reporting Items for Systematic Reviews and Meta-Analysis (PRISMA) statement guidelines (see Supplementary Table S1) [17].

### 2.1. Literature Search

The authors performed a systematic computerised literature search in PubMed, Medline, and Web of Science using the following Boolean search strategy: “(patellar OR patella) AND (tendinopathy OR tendinosis OR “tendon pain” OR tendinitis OR “jumpers knee” OR “jumper´s knee”) AND (treatment OR therapy OR exercise OR training)”. In addition, the search was limited to the following: full-text availability, publication dates: 1 January 1960 to 31 July 2020, language: English, article type: no review. Moreover, the reference lists of the included articles were screened to identify other suitable studies for inclusion in our analysis.

### 2.2. Selection Criteria

We considered studies to be eligible for inclusion in our review if they provided enough relevant information with regard to the PICOS (Population, Interventions, Comparators, Outcomes, Study design) approach. For eligibility, we used the following criteria: (a) Population: participants with a diagnosed (by imaging or palpation technique) patellar overuse injury; (b) Intervention: physical training protocols comprising eccentric, concentric, or isometric strength training; (c) Comparator: active or passive control group (i.e., different physical intervention, no training at all); (d) Outcome: at least one measure of physical or psychological performance; (e) Study design: controlled trials with pre- and post-measures. The exclusion criteria were as follows: (a) participants were not diagnosed by a health professional; (b) intervention was not exercise-based (i.e., ultrasound, injection, dry needling, shockwave); (c) reported data did not allow for calculation (i.e., no central tendency and dispersion measure in the results section or upon request); (d) effects were examined without control condition; (e) assessments did not include a physical or psychological outcome measure; (f) cross-sectional study design. Two independent reviewers (MN, TM) assessed the eligibility of the relevant papers by analysing titles, abstracts, and full texts of the respective articles.

### 2.3. Study Coding

The included studies were coded for the variables listed in Table 1. In our analyses, we focused on different categories of outcome measures. If an eligible article reported multiple variables within one of these categories, we prioritized the most commonly reported outcome for each category in order to reduce heterogeneity between studies. In the case that a study included only similar, but not identical, tests, we selected the test that seemed most relevant with regard to physical or psychological performance outcomes. Jump and reach tests as well as countermovement jumps (with/without use of arms) were considered to be the most important variables of muscle power. Validity and reliability have been shown for both tests [18,19]. Pain at rest was evaluated using the Victorian Institute of Sport Assessment—Patellar tendon (VISA-P) scale or the Visual Analogue Scale (VAS) scale score, whereas pain during exercise was measured using pain during single leg decline squat (SLDS). Validity and reliability have previously been shown for the VISA-P [20], the VAS [21], and the SLDS [22]. Further, we considered the use of training equipment, different types of exercises, the use of closed or open kinetic chain, and the type of underground used. Treatment modality was coded according to the following parameters: training weeks/sessions, number of sets, number of repetitions, exercise duration, and training intensity. If the considered studies did not disclose relevant results, the authors were contacted via email [23,24,25,26,27]. When authors failed to respond to our request, or the requested data was no longer available [24,25], we excluded the respective outcome measure.

### 2.4. Assessment of Methodological Study Quality

The Physiotherapy Evidence Database (PEDro) scale was used to assess the methodological quality of all eligible studies, as well as to quantify the risk of potential bias. The PEDro scale rates internal study validity and the presence of statistical replicable information on a scale from 0 to 10, with ≥6 representing a cut-off score for studies of high quality [28]. Two independent reviewers (MN, TM) assessed the quality of the included studies.

### 2.5. Statistical Analyses

To determine the effectiveness of physical training on physical and psychological parameters in individuals with patella tendinopathy, we calculated the weighted standardized mean difference (SMD = (mean post-test value for the intervention [INT] group minus mean post-test value of the control [CON] group)/pooled variance) using a random-effects meta-analysis model provided by the Review Manager 5.3 software (version 5.3.5, The Nordic Cochrane Centre, Copenhagen, Denmark). From nine studies [14,25,26,27,29,30,31,32,33] that only reported the median and range, we calculated the mean and the standard deviation, in agreement with Hozo et al. [34]. For another study [35] that only stated confidence intervals, we used the formula provided in the Cochrane Handbook of Systematic Reviews of Interventions [36] to calculate the standard deviations. The weighting of the included studies was also performed with the help of Review Manager 5.3 software. In accordance to Cohen, effect size values of 0 ≤ 0.49 indicate small, of 0.50 ≤ 0.79 indicate medium, and of ≥0.80 indicate large effects. In addition, *I*^2^ statistics was used to assess heterogeneity between studies. In agreement with Deeks et al. [37], heterogeneity can be classified as being either trivial (0% ≤ *I*^2^ ≤ 40%), moderate (30% ≤ *I*^2^ ≤ 60%), substantial (50% ≤ *I*^2^ ≤ 90%), or considerable (75% ≤ *I*^2^ ≤ 100%).

## 3. Results

**Figure 1:**
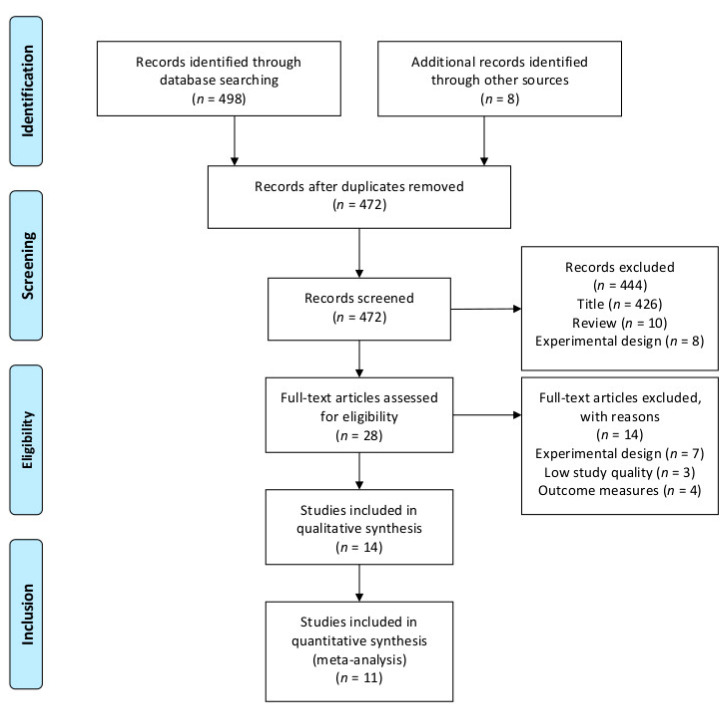
Flowchart illustrating the different phases of the literature search and study selection.

Figure 1 summarizes the process of the systematic literature search, which identified a total of 506 studies. After removing duplicates, screening titles, and excluding ineligible articles, eleven studies remained and were included in our meta-analysis.

### 3.1. Characteristics of the Included Studies

Table 1 displays the characteristics of the eleven included studies. Two studies [23,30] reported data for both physical and psychological variables, eight studies [14,26,27,29,31,32,33,35] reported data for only psychological variables. A total of 277 subjects participated in the eleven trials; 155 of them received eccentric strength training, 42 concentric strength training, 23 isometric strength training, 13 dynamic heavy slow resistance training, 29 sports training, and 20 tendons were surgically treated. The sample size of the included studies ranged from 15 to 43 subjects, and participants had a mean age of 16–32 years. Training periods ranged from 4 to 24 weeks, with the respective training frequencies ranging from 0.5 to 14 sessions per week. Training protocols comprised unilateral/bilateral squats on a decline board/flat surface/unstable surface, leg extensions, leg curls, leg press, as well as static stretching. All eleven studies analysed outcomes of pain assessment (1 × VISA-P, 2 × VAS, 3 × VISA-P/VAS, 1 × VISA-P/SLDS) [14,23,25,26,27,29,30,31,32,33,35]. Three studies reporting physical data examining strength variables (2 × CMJ, 4 × leg strength) [23,25,30].

### 3.2. Methodological Study Quality

The median PEDro score of the included studies was six, which equals the predetermined cut-off value of ≥6 and was achieved by six out of eleven studies (Table 1).

### 3.3. Effects of Physical Training on Physical Parameters in Individuals with Patella Tendinopathy

**Figure 2:**
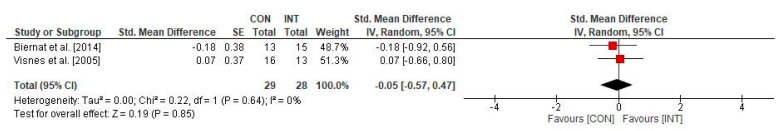
Effects of physical training on physical parameters (i.e., muscle power) in individuals with patella tendinopathy. CI: confidence interval; CON: control group; *df*: degrees of freedom; INT: intervention group; IV: inverse variance; *SE*: standard error; Std.: standard.

Figure 2 shows the effectiveness of physical training on measures of muscle power in individuals with patella tendinopathy. The weighted mean SMD amounted to −0.05 (2 studies; *I^2^* = 0%, *Chi*^2^ = 0.22, *df* = 1, *p* = 0.85), which is indicative of a small-sized effect in favour of the CON group.

### 3.4. Effects of Physical Training on Psychological Parameters in Individuals with Patella Tendinopathy

**Figure 3:**
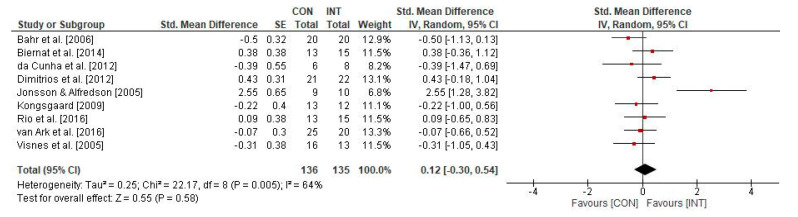
Effects of physical training on psychological parameters (i.e., VISA-P) in individuals with patella tendinopathy. CI: confidence interval; CON: control group; *df*: degrees of freedom; INT: intervention group; IV: inverse variance; *SE*: standard error; Std.: standard.

Figure 3 illustrates the impact of physical training on the psychological measure VISA-P in individuals with patella tendinopathy. The weighted mean SMD yielded 0.12 (9 studies; *I^2^* = 64%, *Chi*^2^ = 22.17, *df* = 8, *p* = 0.005), indicating a small-sized effect and favouring the INT group.

**Figure 4:**
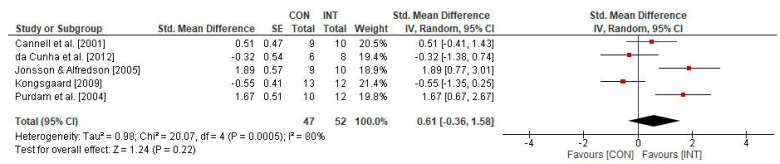
Effects of physical training on psychological parameters (i.e., VAS) in individuals with patella tendinopathy. CI: confidence interval; CON: control group; *df*: degrees of freedom; INT: intervention group; IV: inverse variance; *SE*: standard error; Std.: standard.

The effect of physical training on the psychological measure VAS in individuals with patella tendinopathy is displayed in Figure 4. The weighted mean SMD amounted to 0.61 (5 studies; *I^2^* = 80%, *Chi*^2^ = 20.07, *df* = 4, *p* = 0.22), which indicates a medium-sized effect in favour of the INT group.

## 4. Discussion

To the best of our knowledge, the present systematic review with meta-analysis is the first to examine and quantify the effects of physical training on proxies of physical and psychological parameters in individuals with patella tendinopathy. The analyses of the data of eleven studies revealed mixed results with (i) small-sized but better effects on muscle power for the CON group, (ii) small-sized but better effects on VISA-P for the INT group; and (iii) medium-sized effects on VAS in favour of the INT group.

### 4.1. Effects of Physical Training on Physical Parameters in Individuals with Patella Tendinopathy

Physical training displayed only minimal positive effects on variables of muscle power. Eccentric training intervention, in particular, which had been considered useful in previous reviews [38,39], showed only small effects on muscle power compared to team training. However, it is important to bear in mind that Biernat et al. [30] and Visnes et al. [23] both implemented a very high training volume (two sessions daily) in comparison to other studies. The eccentric training protocol was performed on a decline board, a method which had shown positive results in previous studies. While both studies examined volleyball players, Visnes et al. [13] included both adult males and females and Biernat et al. [30] included adolescent male athletes only. The subjects in the above-mentioned studies also took part in all team practices and matches regardless of pain intensity, therefore resulting in a much higher training load than the CON group, which only took part in team practice. Furthermore, on days with intensive team practice, the subjects were permitted to decide whether or not to take part in the training intervention on their own, which may have led to inconsistent training. Both studies only included subjects with a high burden of symptoms, which may have further affected the impact of the intervention and the volleyball practice. It can be assumed that the high training intensity resulted in an immense burden on the knee joint and the patella, especially as a result of the frequent jumps in volleyball, therefore causing further strain inhibiting the positive effects of training on muscle power.

Overall, a complex, high-intensity physical training intervention should not be combined with intensive team practice and competitive matches. In this regard, further research is required in order to prove the different effects of combined versus single-mode physical exercise interventions on muscle power. Moreover, the investigation of different strength training methods (isometric, eccentric, concentric) in combination with team training may be able to provide insights into the efficacy of therapy.

### 4.2. Effects of Physical Training on Psychological Parameters in Individuals with Patella Tendinopathy

We were able to detect a small-sized effect concerning pain reduction, which was verified by the VISA-P test. Previous reviews [38,39,40] revealed a significant relief of pain and symptoms using eccentric and isometric training in comparison to other treatment methods. In Bahr’s et al. [25] randomized study, it must be considered that the CON group received surgical treatment and, post-operation, the same eccentric intervention as the INT group. It is therefore not possible to ascribe their results to either surgical or conservative intervention. The results of da Cunha et al. [26] must also be interpreted carefully, since both groups received an eccentric training intervention; while the INT group performed the exercises at maximum pain level, the CON group trained without any pain. No significant group differences could be determined, but both groups reported an improvement in the pain and symptoms. Of the studies examined, Jonsson and Alfredson [31] showed the clearest advantages of eccentric training in contrast to concentric training. However, this study was lacking in terms of sample size and study quality, so these outcomes need to be interpreted accordingly. More specifically, this study only included 15 patients and showed a rather low methodological quality because no information was provided for some criteria of the PEDro scale (i.e., concealed allocation; blinding of subjects, therapists, and assessors; adequate follow-up or intention-to-treat analysis). While our systematic review with meta-analysis reported only a medium-sized effect of VAS favouring the INT group, Jonsson and Alfredson [31] showed the most significant improvement of the INT group in comparison to the CON group. Similarly to da Cunha et al. [26], Purdam et al. [32] compared two eccentric exercises with one another; while the CON group performed the exercises on a flat surface, the INT group used a decline board. Therefore, this study cannot make claims pertaining to the effectiveness of eccentric training per se, but only to a variation of eccentric training, which relaxes the calf muscles and in doing so exerts a greater load on the knee extensor muscles. Regarding the time frame of pain reduction, Purdam et al. [32] as well as Jonsson and Alfredson [31] reported a significant pain reduction after twelve weeks in the eccentric group. Further, Bahr et al. [25] did not examine or describe any change in pain reduction before the first testing after twelve weeks. Lastly, da Cunha et al. [26] described an improvement in pain reduction after only eight weeks in both treatment groups—results which are similar to those of Rio et al. [27] and van Ark et al. [29], who found a significant improvement in both treatment groups after four weeks.

In sum, there are slight advantages of eccentric and isometric training compared to other treatment methods in terms of pain management, and these exercises can also be performed using simple equipment. Since all the investigated studies implemented similar intensities and extents of eccentric/isometric training, but none of them combined both methods, it is not possible to draw an adequate conclusion as to what an effective conventional therapy entails. Furthermore, the question whether withdrawing from sports and activities is beneficial for pain and symptom management remains unanswered. The studies presented in this systematic review only vaguely comment on this without describing in detail the intensity/duration of activity, so it is impossible to determine the effect of this variable on the study outcomes.

### 4.3. Limitations

The heterogeneity among the included studies with regards to the implemented training modalities (i.e., eccentric, concentric, or isometric training) is a strong limitation of this systematic review and meta-analysis. Thus, it remains unclear which type of strength training produces the greatest effect on physical and psychological parameters in individuals with patella tendinopathy. By using equal load dimensions (i.e., training duration, frequency, volume), future studies should directly compare the effects of different physical training programs (i.e., eccentric vs. concentric vs. isometric strength exercises) on physical and psychological parameters in individuals with patella tendinopathy. Another limitation is that only active CON groups (i.e., a different type of physical training) were used in the included studies. This circumstance most likely led to the fact that the effects in the INT groups were only small (muscle power and VISA-P) to medium (VAS) compared to the CON groups.

## 5. Conclusions

Unlike previous reviews, the present systematic review and meta-analysis detected only small (muscle power and VISA-P) to medium (VAS) effects among both physical and psychological parameters when comparing eccentric and isometric training protocols to other treatment methods. We therefore conclude that further research is needed to establish the optimal treatment modality in order to reduce pain and symptoms in athletes with patella tendinopathy. Although eccentric and isometric training seem to be useful in pain management, we did not find any study that combined different methods in order to find benefits when used synergistically. Moreover, we were unable to find a study that examined the post-interventional effects on performance in training and competition. The studies included in this review mainly used isolated exercises, such as eccentric leg extension, that were not combined with other treatment methods as training intervention. In addition, only active CON groups were used and compared with the treatment groups. This likely contributed to the fact that only small (muscle power and VISA-P) to medium (VAS) effects were found.

## Figures and Tables

**Table 1:** Included studies examining the effects of physical training on physical and psychological parameters in individuals with patella tendinopathy.

References	No. of Subjects (Tendons); Sex; Age (Mean ± *SD*, or Range); Training Status; Sport	Diagnosis	Groups (Subjects/Tendons); Treatment Type	Treatment Modality: No. of Training Weeks/Sessions; No. of Sets/Reps/Duration Per Exercise; Training Intensity	Test Modality, Outcome Measures	PEDro
van Ark et al. [29]	29 (45); 2 F, 27 M; 23.0 ± 4.7 yrs; sub-elite; volleyball, basketball	Palpation	INT (*n* = 13/20); strength training (isometric quadriceps training, leg extension machine) CON (*n* = 16/25); strength training (concentric quadriceps training, leg extension machine)	4 wk/12 sessions; 5 sets of 45 s hold at 60° flexion at 80% MVIC 4 wk/12 sessions; 4 sets of 8 reps (4 s eccentric, 3 s concentric) at 80% 8 RM	*Physical:* NT *Psychological:* VISA-P (score), SLDS (Numeric Rating Scale 0–0) at pre-/post-intervention	5
Rio et al. [27]	20 (28); 2 F, 18 M; ≥16 yrs; elite and sub-elite; volleyball, basketball	Imaging, SLDS test	INT (*n* = 10/15); strength training (isometric quadriceps training, leg extension machine) CON (*n* = 10/13); strength training (concentric quadriceps training, leg extension machine)	4 wk/12 sessions; 5 sets of 45 s hold at 60° flexion at 80% MVIC 4 wk/12 sessions; 4 sets of 8 reps (4 s eccentric, 3 s concentric) at 80% 8 RM	*Physical:* NT *Psychological:* VISA-P (score) at pre-/post-intervention	6
Biernat et al. [30]	28 (NR); M; 17.0 ± 0.1 yrs; recreational; volleyball	Imaging	INT (*n* = 15/NR); volleyball training + strength exercises (eccentric) CON (*n* = 13/NR); volleyball training only	4 wk/daily; 3 sets of 15 reps on decline board followed by 20 wk/daily; 3 sets of 15 reps on unstable decline board 24 wk/12 sessions; 5 sets of 45 s hold at 60° flexion at 80% MVIC	*Physical:* Isokinetic leg strength; CMJ (cm, W) *Psychological:* VISA-P (score) at pre-/mid-/post-intervention	5
Dimitrios et al. [35]	43 (NR); 12 F, 31 M; 27 ± 5 yrs; recreational; NR	Palpation, SLDS test	INT (*n* = 22/NR); strength (eccentric) and stretching (static) exercises CON (*n* = 21/NR); strength (eccentric) exercises	4 wk/20 sessions; 3 sets of 15 reps on decline board, weight increased when pain-free, static quadriceps/hamstrings stretching before and after the eccentric training, 4 exercises of 30 s 4 wk/20 sessions, 3 sets of 15 reps on decline board, weight increased when pain-free	*Physical:* NT *Psychological:* VISA-P (score) at pre-/post-intervention	5
da Cunha et al. [26]	17 (14); 3 F, 14 M; 25 ± 8 yrs; recreational; volleyball, soccer, athletics, basketball, handball, capoeira, jiu jitsu, triathlon, skating	Imaging	INT (*n* = 10/8); strength (eccentric) training, maximum pain CON (*n* = 7/6); strength (eccentric) training, pain-free	12 wk/36 sessions; 3 sets of 15 reps, weight increased when reps possible, pain mandatory 12 wk/36 sessions; 3 sets of 15 reps, weight increased when pain-free	*Physical:* NT *Psychological:* VISA-P (score), VAS (0–10) at pre-/mid-/post-intervention	7
Kongsgaard et al. [14]	25 (25); 25 M; 32 ± 8 yrs; recreational; running, soccer, basketball, floorball, handball	Palpation, imaging	INT (*n* = 12/12); strength (eccentric) training CON I (*n* = 13/13); strength (dynamic) trainingCON II (*n* = 12/12); peritendinous corticosteroid injections	12 wk/168 sessions; 3 sets of 15 reps on 25° decline board, pain-acceptable, weight increased when pain diminished 12 wk/36 sessions; 4 sets of 6–15 reps, pain-acceptable ultrasound-guided injections of 1 mL of 40 mg/mL methylprednisolon	*Physical:* MVIC *Psychological:* VISA-P (score), VAS (0–100) at pre-/post-intervention and 6 months post-intervention	6
Bahr et al. [25]	35 (40); 5 F, 30 M; 30 ± 8 yrs; recreational; running, soccer, handball, martial arts	Palpation, imaging	INT (*n* = NR/20); eccentric decline squat, sports from wk 8+ CON (*n* = NR/20); surgical treatment, sports from wk 8+	12 wk; 9.3 ± 4.1 sessions per wk; 3 sets of 15 reps on 25° decline board to 90° knee flexion, moderate pain mandatory (VAS = 4/5), weight increased when VAS < 3 12 wk post-operative training; sessions increased weekly; 10.1 ± 4.3 sessions per wk; wk 1: isometric quadriceps exercises, wk 2: adding walking, wk 3: adding cycling and high squats, wk 4: adding step-ups to a low (5–6 cm) step, wk 5: step-downs from a low (5–6 cm) step, wk 6: adding eccentric squat training similar to INT group but without any pain	*Physical:* Leg press strength (kg); CMJ (cm) at pre-intervention only *Psychological:* VISA-P (score) at pre-/post-intervention and 6 and 12 months post-intervention	7
Jonsson and Alfredson [31]	15 (19); 2 F, 13 M; 25 ± 9 yrs; recreational; running, soccer, basketball, floorball, handball	Palpation, imaging	INT (*n* = 8/10); strength (eccentric) training CON (*n* = 7/9); strength (concentric) training	12 wk/168 sessions; 3 sets of 15 reps on 25° decline board, pain mandatory, weight increased when reps not painful 12 wk/168 sessions; 3 sets of 15 reps concentric knee extension from 70° knee flexion on 25° decline board, pain mandatory	*Physical:* NT *Psychological:* VISA-P (score), VAS (0–100) at pre-/post-intervention	4
Visnes et al. [23]	29 (29); 10 F, 19 M; 27 ± 4 yrs; elite; volleyball	Palpation	INT (*n* = 13/13); strength (eccentric) training CON (*n* = 16/16); volleyball training only	12 wk/168 sessions; 3 sets of 15 reps on 25° decline board to 90° knee flexion, 2 s eccentric phase, weight increased when VAS < 5 volleyball training as usual without information on training load	*Physical:* CMJ (cm), SJ (cm) *Psychological:* VISA-P (score) at pre-/post-intervention and 6 and 24 wk post-intervention	7
Purdam et al. [32]	17 (22); 4 F, 13 M; 25 yrs; recreational; floorball, soccer, volleyball, running, ice hockey, high jump, skiing	Palpation, imaging	INT (*n* = 8/12); strength (eccentric) training, decline squat CON (*n* = 9/10); strength (eccentric) training, flat-surface squat	12 wk/168 sessions; 3 sets of 15 reps on 25° decline board to 90° knee flexion, some pain mandatory, weight increased when reps not painful 12 wk/168 sessions; 3 sets of 15 reps on flat surface to 90° knee flexion, some pain mandatory, weight increased when reps not painful	*Physical:* NT *Psychological:* VAS (0–100) at pre-/post-intervention	5
Cannell et al. [33]	19 (NR); 6 F, 13 M; 26 ± 7 yrs; recreational; basketball, soccer, running, volleyball, tennis, squash, rowing, American football, gymnastics	Palpation	INT (*n* = 10/NR); progressive strength (eccentric) training, drop squats CON (*n* = 9/NR); progressive strength (concentric) training, leg extension/curl	12 wk/60 sessions; 3 sets of 20 reps, pain mandatory, weight increased when reps not painful; activity (running) added when reps not painful 12 wk/60 sessions; 3 sets of 10 reps with 5 kg each leg extension/leg curl exercise, weight increased when reps not painful; activity (running) added when reps not painful	*Physical:* Isokinetic leg strength *Psychological:* VAS (0–10) at pre-/mid-/post-intervention	7

CMJ: countermovement jump; CON: control group; F: female; INT: intervention group; PEDro: Physiotherapy Evidence Database scale; M: male; MVIC: maximum voluntary isometric contraction; NR: not reported; NT: not tested; RM: repetition maximum; SJ: squat jump; SLDS: single leg decline squat; VAS: visual analogue scale; VISA-P: Victorian Institute of Sport Assessment–Patellar tendon scale; wk: week; yrs: years.

## Data Availability

The data presented in this study are available on request from the corresponding author.

## Supplementary Materials

The following are available online at https://www.mdpi.com/2075-4663/9/1/12/s1, Table S1: PRISMA Checklist: transparent reporting of systematic reviews and meta-analyses.
