# Erratum: Niering, M.; Muehlbauer, T. Effects of Physical Training on Physical and Psychological Parameters in Individuals with Patella Tendinopathy: A Systematic Review and Meta-Analysis. *Sports* 2021, *9*, 12

**DOI:** 10.3390/sports9110151

**Published:** 2021-11-09

**Authors:** Marc Niering, Thomas Muehlbauer

**Affiliations:** 1 Department of Health and Social Affairs, FHM Hannover—University of Applied Sciences, 30163 Hannover, Germany; 2 Division of Movement and Training Sciences/Biomechanics of Sport, University of Duisburg-Essen, 45141 Essen, Germany; thomas.muehlbauer@uni-due.de

The authors would like to correct an error in the name of the condition in the recently published paper [1]. The term “Patella Tendon Myopathy”, which was used in the title as well as in the text, will be replaced by the correct term “Patella Tendinopathy”. This term corresponds to the common wording “jumper’s knee” and describes a degenerative change in the patella tendon [2]. The original article has been updated and entitled as follows: “Effects of Physical Training on Physical and Psychological Parameters in Individuals with Patella Tendinopathy: A Systematic Review and Meta-Analysis”.
